# Surgical intervention for anorectal fistula

**DOI:** 10.1590/S1516-31802011000200014

**Published:** 2011-03-03

## Abstract

**BACKGROUND:**

Surgery for anorectal fistula may result in recurrence, or impairment of continence. The ideal treatment for anorectal fistulae should be associated with low recurrence rates, minimal incontinence and good quality of life.

**OBJECTIVE:**

To assess the efficacy and morbidity of operative procedures for chronic anal fistula, primary outcomes being recurrence and incontinence.

**CRITERIA FOR CONSIDERING STUDIES FOR THIS REVIEW:**

The following databases were searched: EMBASE (Webspirs 5.1, Silver Platter version 2.0, 1950–2009); Medline (Webspirs 5.1, Silver Platter version 2.0, 1950–2009); The Cochrane Central Register of Controlled Trials (2009 issue 4)and the IndMed ( Indian Medline, www.indmed.nic.in) database. We restricted our search to the English literature. The Indian Journal of Surgery was electronically searched (issues between 2003 and vol 71, Oct 2009). We also searched all primary trial registers (Indian, Australian, Chinese, WHO, ISRCTN and American).

**SELECTION CRITERIA:**

Randomised controlled trials comparing operative procedures for anorectal fistulae were considered. Non randomised trials and cohort studies were examined where data on recurrence and function were available.

**DATA COLLECTION AND ANALYSIS:**

Two reviewers (TJ and BP) independently selected the trials for inclusion in the review. Disagreements were solved by discussion. Where disagreement persisted and published results made data extraction difficult, we obtained clarification from the authors. REVMAN 5 was used for statistical analysis. Quality of the trials were assessed and allowances made for subgroup analysis and prevention of publication bias, using funnel plots if needed.

**MAIN RESULTS:**

Ten randomized controlled trials were available for analysis. The quality of included studies was adequate, though in some trials the numbers were small and they were inadequately powered for equivalence or to detect significant differences. Comparisons were made between various modalities of treatments. There were no significant difference in recurrence rates or incontinence rates in any of the studied comparisons except in the case of advancement flaps. There were more recurrences in the glue plus flap group, a significant difference that favoured the flap only technique. It was also noted that Fibrin glue and advancement flap procedures report low incontinence rates. In the review of literature of non-randomized trials, most trials on fibrin glue indicate good healing in simple fistulae with low incontinence rates.

**AUTHORS' CONCLUSIONS:**

There are very few randomized controlled trials comparing the various modalities of surgery for fistula in ano. While post operative pain, time to healing and discharge from hospital affect quality of life, recurrence and incontinence are the most important. As it turns out, there seems to be no major difference between the various techniques used as far as recurrence rates are concerned.

**Sarhan Sydney Saad, MD. PhD.** Adjunct Professor of the Discipline of Surgical Gastroenterology, Universidade Federal de São Paulo — Escola Paulista de Medicina (Unifesp-EPM), São Paulo, Brazil. Titular Member of the Brazilian Society of Coloproctology (Sociedade Brasileira de Coloproctologia, SBCP), Rio de Janeiro, Brazil.

## PLAIN LANGUAGE SUMMARY

Anorectal fistula is a common surgical problem. The anorectal fistula can be treated by various surgical options. The common surgical options for rectal fistulae are

- to lay open the fistula tract (fistulotomy), or
- to pass a seton (a thread, wire or tube that stimulates the body to extrude it and ultimately heal the fistula), or
- to primarily remove the fistula (fistulectomy) and repair the defect in the muscle and the anus with an anorectal myo-mucosal advancement flap.

Other treatment modalities used less frequently include fibrin glue and ayurvedic drugs incorporated into setons. The optimal surgical treatment for anorectal fistulae is associated with low recurrence rates, minimal incontinence and a good quality of life.

The reviewers identified 10 trials that compare various fistula treatments against one another. There are various parameters that these procedures can be compared on, but we looked at the two most important ones, recurrence (the numbers who got the disease again) and incontinence (a worsening in the ability to control rectal content).

In the trials that were compared, there was no significant difference between the various comparisons for the disease to recur. However the trials on fibrin glue, as well as data from non randomised trials show that incontinence is less, probably as there is no surgical disruption of the anal muscle.

There is a paucity of good quality data that compares various types of operative treatment for anorectal fistula and there is scope for further trials in the area.

## COMMENTS

The best medical scientific evidence currently accepted is provided by systematic reviews and meta-analyses. These tools make it possible to group published studies with appropriate methodology, thereby achieving an adequate number of patients in each arm of a medical study and enabling comparison between different treatment methods.

In the systematic review in question, the first point to which the authors draw attention is the small number of published papers with appropriate methodology that they found in the literature, i.e. only 10 randomized controlled prospective studies. Study selection was done through analysis by two reviewers, and any differences of opinion were resolved by reaching a consensus. The main end points of this review were the incidence of recurrence and fecal incontinence. The authors did not find any results that would suggest the existence of differences in the methods proposed for surgical treatment of anorectal fistula, except in comparing the use of glue in association with a flap versus the use of a flap alone; the latter had better results in terms of recurrence. The use of glue and a mucous flap led to a low rate of incontinence. The authors also included some non-randomized studies and concluded that fibrin glue can be used safely in simple fistulas.

From the results presented, it can clearly be seen that the literature is deficient in appropriate published papers on this subject. There is a need to produce scientific evidence through good-quality contributions to the literature. It is possible that the differences are very small and can only be seen with large numbers of patients in each group of patients that is to be compared. The results from this review allow surgeons to choose perianal fistula treatment methods in which they have greater experience, because there is no evidence so far in the literature to show that any method is superior to any other.
